# Exploring the Utilization of Activated Volcanic Ash as a Substitute for Portland Cement in Mortar Formulation: A Thorough Experimental Investigation

**DOI:** 10.3390/ma17051123

**Published:** 2024-02-29

**Authors:** Andrés Játiva, Miren Etxeberria

**Affiliations:** Department of Civil and Environmental Engineering, Universitat Politècnica de Catalunya (UPC-Barcelona TECH), 08034 Barcelona, Spain; andres.jativa@upc.edu

**Keywords:** volcanic ash, hybrid cement mortars, alkali activation, calcination compressive strength, physical properties

## Abstract

The manufacture of natural pozzolans as cement products is economically affordable and contributes to CO_2_ mitigation in the cement-based materials industry. Through two experimental stages, this study evaluates the feasibility of using volcanic ash (VA) to partially substitute portland cement (PC) in mortar production. In Stage 1, the effectiveness of different activation methods, such as calcination, alkali activation, and lime addition, in enhancing VA reactivity was assessed when the mortars were produced using 35% VA. The compressive strength (f_cm_) and physical properties of the mortars produced were determined at 7 and 28 days and compared with those of mortars without activated VA. In Stage 2, the most effective treatments obtained from Stage 1 were applied to produce mortars with 50% and 75% of VA replacements, focusing on their physical and mechanical properties. The findings revealed promising results, particularly when mortars were produced with up to 50% calcined VA (CVA) at 700 °C and 20 wt% lime addition, reaching a higher f_cm_ than 45 MPa. Chemical activation with 2% CaCl or 1% NSi enhanced early-age strength in 35% VA-based mortars. Additionally, NSi-activated CVA-lime-based mortar at 50% VA achieved a notable f_cm_ of 40 MPa at 28 days. Even mortars with 75% VA replacement achieved an adequate compressive strength of 33MPa at 28 days. This study determined that VA-based mortars have the potential for construction applications.

## 1. Introduction

Constant population growth in urban areas causes an increase in industrial construction activity and, consequently, cement demand, which results in environmental impacts, making it necessary to find more sustainable alternative solutions [1]. The available natural pozzolan reserves are plentiful, although they are localized resources, their manufacture is economically more affordable and contributes to CO_2_ mitigation in the cement-based materials industry [2]. Natural pozzolan of Volcanic ash (VA) [3] with a chemical composition and amorphous atomic structure is rich in aluminosilicates, making it a suitable material as a substitute for portland cement (PC) clinker as a binder in cement production [4]. However, in practice, substitution levels do not usually exceed 35% by weight because their reactivity is slow, especially at early ages [5,6,7,8]. The ability to increase pozzolanic properties and reactivity using mechanical, thermal, and chemical methods, including alkali activation, has been studied [9,10,11,12]. Moreover, in recent research, alternative cements have been developed that combine PC’s advantages with the alkali activation of VA, creating a long-lasting and eco-friendly material. Based on various studies, this approach has yielded encouraging results [13,14,15].

Several studies have indicated that calcinating VA to at least 500 °C can alter its mineralogical structure, making it more amorphous and reactive [16,17]. Furthermore, this process removes organic matter from the VA as well as reducing its porosity and weight by eliminating water content, thus enhancing its ability to react and bond with other materials [18].

VA is a semicrystalline material with less reactive components, such as CaO, SiO_2,_ and Al_2_O_3_. Consequently, commonly used chemical alkaline activators that include sodium silicate (Na_2_SiO_3_, NSi) and sodium hydroxide (NaOH, NH) [19] can be used to increase reactivity. To increase the activity of binders, many researchers have used NaOH alone or mixed it with silicates as activators [20,21,22,23]. The use of VA in hybrid cement promotes the hydrolysis of aluminosilicate bonds, due to the alkaline environment provided by PC. During the early stages of cement hydration, high alkalinity favours the formation of N-A-S-H/(N, C)-A-S-H gels in detriment to C-S-H gels and inhibits portlandite formation [24]. Additionally, the heat generated by cement hydration from the PC clinker enhances this activation.

The solid activators sodium sulphate (Na_2_SO_4_, NS), sodium carbonate (Na_2_CO_3_, NC), and calcium chloride (CaCl_2_, CaCl) are attractive for industrial applications because their production is cheaper and easier to handle than that of aqueous silicate and hydroxide solutions. In addition, they are available and cause negligible CO_2_ emissions compared to common activators (hydroxides and silicates) [25]. However, limited research has been conducted to analyse their applicability, focusing mainly on paste mixtures [26,27,28]. NC activators are considered “weak” due to their low pH in a solution. However, carbonates often activate calcium-rich silicate precursors, such as blast furnace slag and VA + PC. Bernal et al. (2015) [29] concluded that NC-activated blast furnace slag binders undergo a reaction mechanism similar to that of NH, resulting in the formation of a hydrated calcium aluminosilicate (C-A-S-H)-type gel with high strength and excellent durability after completion of the carbonate ion reaction.

The NS activator is also a low-pH activator, similar to carbonate, but it produces a weaker and more porous structure than the NC activator. When NS reacts with Ca(OH)_2_, it creates an alkaline environment that accelerates the dissolution of active alumina and silica in the cement–pozzolan–water system. Studies have revealed that cementitious materials with fly ash and slag can benefit from sulphate-forming ettringite, which enhances their mechanical properties [30,31]. NS-activated mortars exhibit less shrinkage than silicate-activated mortars because of the creation of more ettringite crystals [32]. A study by Vehmas et al. [33], determined that CaCl provided valuable results confirming that it is a powerful accelerator in the initial stages of hydration as a powerful accelerator for hydration. However, it is not widely used owing to its potential to accelerate steel corrosion.

Lime has been successfully evaluated as a compositional corrector of VA to reduce the need for PC in hydraulic pozzolanic cement. Combining volcanic tuff with 35% slaked lime created high-strength mortars of up to 22 MPa [34] and improved cement durability, offering resistance to acids and sulphates [35,36,37], thermal stability [38], and carbonation [39,40].

This study aimed to assess the applicability of VA originating from northern Ecuador. Ecuador has 55 volcanoes formed with natural pozzolans, which potentially can be used to make sustainable construction materials [41]. Natural pozzolans deposits are easily accessible and can be naturally mined thus having a better cost–benefit ratio than the traditional quarrying method commonly used for clay mining. Additionally, in countries with high economic growth, these deposits can have significant commercial value for the cement industry because the VA can be used to produce blended cement [2,42].

The VA was employed as a partial substitute for PC in mortar production. Two experimental stages (Stages 1 and 2) were conducted. In Stage 1, the effectiveness of different activation methods in enhancing VA reactivity was assessed when the mortars were produced using 35% VA. Calcination of VA, alkali activation (AA), and lime (L) addition were used as activation methods. Calcination temperatures between 500–900 °C were employed. Alkaline activators (AA) Na_2_SiO_3_ (NSi), CaCl_2_ (CaCl), Na_2_SO_4_ (NS), and Na_2_CO_3_ (NC) were tested at different dosages to identify the most effective ones. 10%, 20%, and 30% of lime (L) related to VA weight were added to correct the CaO deficit of VA. The compressive strength and physical properties of the mortars produced after 7 and 28 days were determined, analysed and compared with the values obtained by the mortar produced with no activated VA. In Stage 2, the most effective treatments identified in Stage 1 were employed for producing mortar with 50% and 75% VA, and their physical and mechanical performance were also evaluated. All the mortar mixtures were subjected to standard curing conditions at 22 °C and 95% relative humidity.

## 2. Materials and Methods

### 2.1. Materials

All mortars were produced using Type CEM I 52.5R, ordinary PC, with a density of 3.1 kg/dm^3^. The chemical compositions are listed in Table 1. Fresh drinking water from the city of Barcelona was also used in all mortar mixtures and normalised sand, following the specification ASTM C778 “Specification for Standard Sand” [43]. The grain sizes were in the range of 0.08–2.00 mm. In addition, a superplasticiser (SP) based on modified polymers in an aqueous solution was employed in the mortars required to be compactable.

#### 2.1.1. Ecuadorian Volcanic Ash (VA)

The VA used in this study was supplied by the Otavalo region in northern Ecuador. The chemical composition was determined using Wavelength-Dispersive X-ray Fluorescence Spectrometer model PERFORM’X (Thermo Fisher, Waltham, MA, USA) X-ray fluorescence (XRF) (Table 1). The main components of VA were silica (SiO_2_) and alumina (Al_2_O_3_). VA also contained minor proportions of other pozzolanic oxides, such as ferric oxide (Fe_2_O_3_) and magnesium oxide (MgO), frequently found in acidic rocks. The VA used in this study was similar to the basalt-andesitic VA reported in several studies [16,21,44], with 54.5–60% silica contents and 14–17% alumina [45]. The sum of principal oxides (SiO_2_ + Al_2_O_3_ + Fe_2_O_3_) was more than 70% (Table 2), the minimum percentage to categorise it as a pozzolanic material for mortar production. The presence of CaO 6.30% in VA was higher than other VAs reported [16,23,46,47]. It could be favourable, as calcium could be responsible for the hydraulic cementitious properties of the material and, consequently, the strength development of mortars [7,48]. In addition, Table 1 shows that VA presented a low loss of ignition, probably due to the limited content of zeolite or clay-derived minerals, organic matter, and hydroxides [9]. The Loss on Ignition was determined by exposing the VA to a temperature of 1000 °C for 1 h. The SiO_2_/Al_2_O_3_ molar ratio was 3.3, ideal for producing favourable properties in alkali-activated binders [49].

The moisture content shown in Table 2 was determined by drying the VA in an oven at 100 °C for 24 h. The strength activity index (SAI) of VA was determined and validated to be higher than 75%, which is the minimum required value. The specific surface area of VA was determined using the Brunauer–Emmett–Teller (BET) method on a Micromeritics Flowsorb ASAP 2020 instrument (Micromeritics, Norcross, GA, USA), which involves the physical adsorption of N_2_ gas on the surface of the VA particles. The BET surface area of the VA was 1.65 m^2^/g. Previous reports on the specific surface area of VA ranged from smaller than 2.5 m^2^/g [23,51,52] to 10 m^2^/g [53]. The apparent density of the VA was 2.73 kg/m^3^. The VA was dried at 105 ± 5 °C for 24 h before its use.

**Figure 1:**
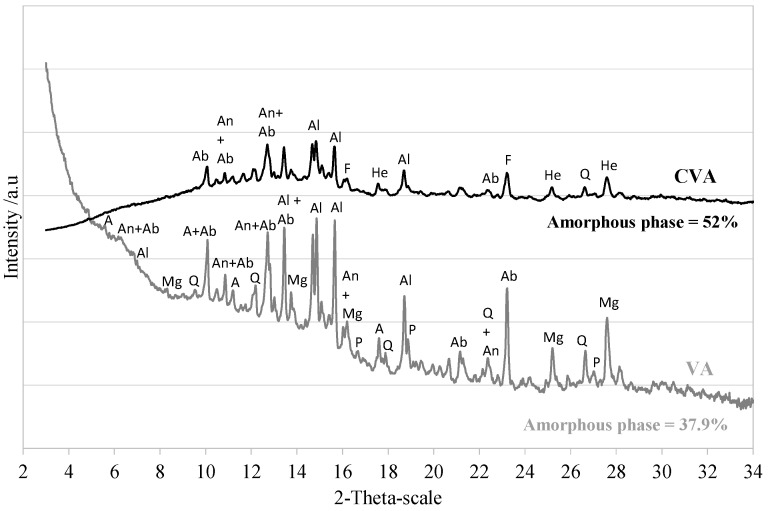
Mineralogical composition of untreated VA VA and CVA at 700 °C (A: Antigorite; Q: Quartz; Al: Alite; Ab: Albite; An: Anorthite; Mg: Magnesium ferrite; P: Periclase; F: Forsterite; He: Hematite).

Figure 1 shows the crystallography and mineral composition of the VA used in this study. The X-ray diffraction (XRD) pattern of the VA was obtained using a Bruker D8 forward diffractometer operating with Mo radiation (Mo Kα1) and 2θ scanning ranging from 3° to 30° covered over two hours. Amorphous silica is responsible for the pozzolanic activity of the sample, whereas the amount of quartzite silica does not have pozzolanic properties [54]. These parameters provide an idea of VA quality. An experimental procedure was used to quantify the amorphous content in VA based on Rietveld refinement using the corundum (Al_2_O_3_) standard reference material (ALUMINA NIST SRM 676-a) and the High Score Plus ^®^ software (v4.9a). The standard was chosen based on having similar X-ray absorption to the sample. The measurement was conducted on the fine powder sample with approximately 20% by weight of corundum alumina standard. The hump of the diffractogram between 10° and 27° 2θ indicates that VA also has a non-diffractive portion. It was determined that it had 38% amorphous material. Several studies have reported similar amorphous and vitreous phases that result in VA [47,55]. According to several researchers [21,56], a minimum of 20% amorphous phase content should allow the VA to be efficiently activated and achieve cementing properties. The crystalline fraction of VA was mainly composed of 45.4% sodium-calcium feldspars or plagioclase (anorthite series). In addition, some minerals, including quartz (4–5%), montmorillonite (2.3%), iron and magnesium oxides, magnetite (7%), and hornblende-magnesium 10%, were found.

The leachable metal values for VA were determined using the EN12457-2 [57] leaching test (Table 3). The eluate obtained after the test procedure was analysed using an inductively coupled plasma collision cell mass. All leaching tests were performed in duplicate. The results were compared with the regulatory limits issued by the European Council (2003) [58] and were classified as stable non-reactive solids, as the Cr concentration reached 6.51 mg/kg.

#### 2.1.2. Calcined VA (CVA)

VA was calcinated (CVA) at 500, 700, 800 and 900 °C for 1 h. The heating rate was 5 °C for 1 h followed by rapidly cooling to room temperature (25 °C ± 1).

#### 2.1.3. Alkaline Activators (AA)

Four types of AA were employed: (1) sodium silicate (Na_2_SiO_3_ (NSi), 99.0% purity) with a composition of 26.4% SiO_2_, 8.0% Na_2_O, 65.5% H_2_O, and a modulus of 3.35; (2) sodium sulphate anhydrous (Na_2_SO_4_ (NS), 99.0% purity, powder); (3) sodium carbonate (Na_2_CO_3_ (NC), 99.0% purity, powder), and (4) calcium chloride anhydrous (CaCl_2_ (CaCl), 99.0% purity, powder).

#### 2.1.4. Lime (L)

Lime (Ca(OH)_2_) powder type CL-80 S (UNE-EN-459) was used to offset the calcium deficiencies of the VA in the mortar mixtures. The chemical composition of lime is listed in Table 1. The apparent density was 400 kg/m^3^.

### 2.2. Experimental Programme: Production of Mortars Test Procedure

The experiment was conducted in two stages (Stages 1 and 2). In Stage 1, mortars were produced using 35% VA to replace PC. Five experimental phases were conducted to analyse the influence of the VA activation methods on the properties of the mortar. The results were compared to those of the mortar produced with the untreated VA. The best activation methods were identified and applied in Stage 2. In Stage 2, the use of high amounts of VA (50% and 75%) in activated hybrid cement mortars was produced and analysed, then compared to the mortars made with untreated VA binders.

All mortar mixtures were produced according to ASTM C109/C109M standards [59], using a 1:2.75 binder-to-sand (B/S) weight ratio and a fixed water-to-binder ratio (W/B) of 0.48. When needed a superplasticiser (SP) content by binder weight percentage was used to maintain a fluidity of 170 ± 5 mm, in line with ASTM C109 standards. In both Stage 1 and Stage 2, solid binder components (VA, CVA, PC, and L) in different percentages were used under dry conditions for mortar production. After the homogeneous mixing of the solids, water was slowly added. For the alkali-activated mortars, the AA solution which had mixed with water, was slowly added to the solid materials and mixed.

The fresh mortar mixes were placed in standard 40 mm plastic cube moulds and compacted using a vibrating table. The samples were then wrapped in plastic film and kept under high relative humidity conditions (RH > 95%) and a temperature of 22 ± 1 °C in a humidity chamber for 24 h. After demoulding, the samples were maintained under the same curing regime until their mechanical and physical properties were tested at 7 and 28 days.

The physical properties of dry bulk density, porosity, and water absorption for all produced mortars were determined at 7 and 28 days following ASTM C642 “Standard for Density, Absorption, and Voids in Hardened Concrete” [60]. Three 40 mm test cubes were used for each mortar type to obtain the mean values.

The compressive strength (f_cm_) of the mortars was determined using an automatic compression-testing machine. A hydraulic press was employed with a press movement speed of 900 N/s for the test mortars. Three 40 mm cubic specimens were used to measure the f_cm_, which was tested at 7 and 28 days following ASTM C109/C109 M standards [59].

## 3. Stage 1 Mix Proportions. Results and Discussion

### 3.1. Mix Proportions of Mortar with 35% VA

Table 4 describes all the mix proportions produced in Stage 1. All mortars were made using 35% VAs and 65% PC (by weight). In addition, five activation phases were conducted to analyse the VA activity improvement and mortar properties.

- Phase 1.1: Mortar produced with calcined VA (CVA)

The mortar was produced using 35% CVA (CVA35). The VA was calcined at four different temperatures (500, 700, 800, and 900 °C) for 1 h.

- Phase 1.2: Mortar produced using alkali activators (AA)

As shown in Table 4, 14 mortar mixtures were created by employing varying concentrations of four different activators: NSi, CaCl, NS, and NC. The NSi and CaCl were used in three different concentrations, and NS and NC in two.

The AA concentrations are expressed as the % of the binder weight. Different concentrations of NSi, ranging from 0.5% to 2% by weight (wt%), were employed based on various researchers’ findings [13,61]. The water content of this AA was considered to be mixing water. Dry activators NC, NS, and CaCl were used at 2% and 4% for binder weight. In addition, the CaCl activator was also used in 1 wt% [17,32,62]. Dry AA was adequately dissolved in water before being added to the dry binder for mortar production. SP was only used to improve the workability of the alkali-activated mortar mixtures containing 4 wt% of CaCl, NC, and NS. SP was added to the mixer as the latest component after mixing the AA solution (AA + water) with the sand and binder, followed by further mixing. The mixtures were identified as VA35-AAx, where AA is the type of activator (NSi, NC, NS, or CaCl), and x is the dosage employed (%). For instance, VA35–NSi0.5 refers to the mortar with 35% VA and 0.5 wt% NSi by weight concerning the entire binder.

- Phase 1.3: Mortar produced using lime (L) as a corrector

Mortars were produced using L to partially substitute the VA with 10, 20 and 30% (by weight). The binder of mortars was composed of 35% VA + 65% PC. According to various studies, a 10–20 wt% replacement is usually employed as a corrector in mortar production [63,64]. The mixtures were identified as VA35-Ly, where y represents the percentage of L.

- Phase 1.4: Mortar produced using CVA and lime as a corrector

The mortars were produced using CVA (optimal calcined VA, defined in Phase 1.1) and lime (optimal % defined in Phase 1.3). The mixtures were identified as CVA35-Ly, where y represents the percentage of L.

- Phase 1.5: Mortar produced using CVA, lime as corrector and alkali activator

Mortars were produced using the most effective AA determined in Phase 1.2 and lime (optimal % defined in Phase 1.3). The mixtures were identified as AAz-CVA35-Ly, where AA represents the alkaline activator, z indicates the %AA, and y denotes the %L replacement.

### 3.2. Results and Discussion

#### 3.2.1. Compressive Strength (f_cm_)

The effectiveness of the treatment methods, including calcination, AA, and lime addition, in enhancing the reactivity of VA utilised in mortars with 35% VA substitution was evaluated by determining the compressive strength. Table 5 shows the mean compressive strength values (f_cm_) of the mortars produced in Stage 1, and the following sections provide a comprehensive analysis of the impact of the treatment methods on f_cm_.

- Phase 1.1: Mortar produced with calcined VA

Table 5 shows that the mortar produced with CVA calcined at 700 °C for 1 h achieved the highest f_cm_ among the mortars made using CVA materials. The CVA35-700 mortar reached 31 and 45 MPa of f_cm_ values at 7 and 28 days, respectively. In addition, CVA35-700 achieved a higher strength than VA35, with an f_cm_ strength of 29.6 and 37.6 MPa at 7 and 28 days, respectively.

To understand the strength improvement of CVA35-700, the mineral composition of the calcined CVA at 700 °C was analysed by XRD (see Figure 1). In addition, it shows that the calcination process decreased peak intensities when compared to the untreated VA. Moreover, the broad hump observed between 10° and 30° 2*θ* indicated an increase in the amorphous phase [65], a fact confirmed by the Rietveld method for up to 52% amorphous content. New minerals emerged, such as forsterite and haematite, which are related to recrystallising minerals rich in iron and magnesium oxides [66,67].

**Figure 2:**
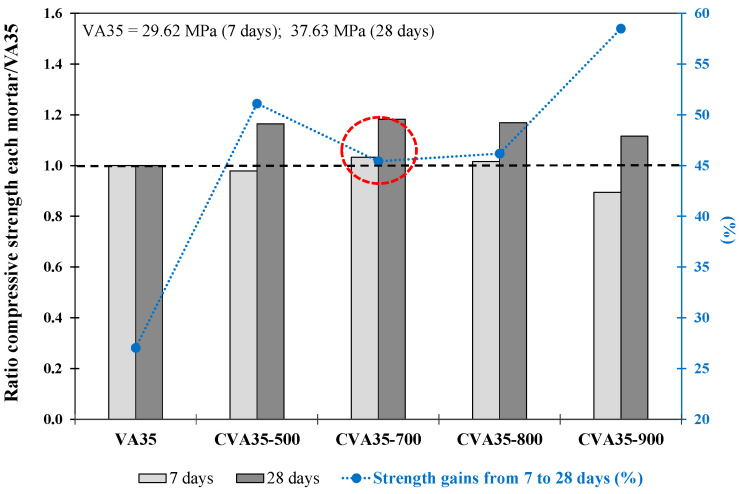
The ratio between compressive strength at 7 and 28 days of each mortar with respect to VA35 mortar. Increase in strength from 7 to 28 days (%) for CVA-based mortars. The red circle describes the optimum mixture

Figure 2 shows the f_cm_ ratio in the mortar produced using 35% CVA at different temperatures (500, 700, 800, and 900 °C) compared with VA35 at 7 and 28 days. The % of strength gain of each mortar from 7 to 28 d is also included.

The CVA35-700 mortar achieved a 19.1% higher f_cm_ at 28 d than the VA35 mortar (marked in red circle). This improvement in compressive strength was due to an increase in the amorphous phase content of the CVA. This fact could also be attributed to the thermal activation of certain minerals initially present in the VA, such as dehydroxylated montmorillonite (between 600 °C and 700 °C) or activated silica around andesite grains. However, calcination at 800 °C and 900 °C reduced reactivity, possibly owing to changes in mineralogical properties during calcination, thereby increasing the degree of crystallinity and peak intensity [68,69]. In addition, while the mortars produced with CVA reached a strength gain (%) from 7 to 28 d between 45% and 58%, the VA35 mortar achieved a strength increase of 27%.

- Phase 1.2: Mortar produced with alkali activator

**Figure 3:**
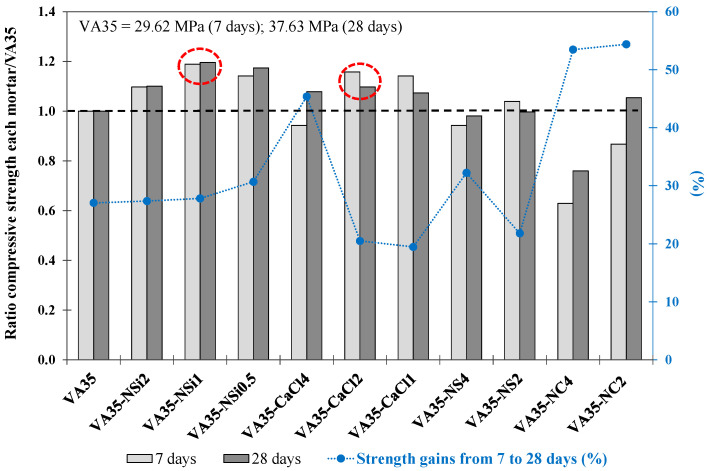
The ratio between compressive strength at 7 and 28 days for each mortar compared to VA35 mortar. Increase in strength from 7 to 28 days (%) of VA-based mortars produced with AA activators. The red circles describe the optimum mixtures

Table 5 describes the f_cm_ values obtained at 7 d and 28 d for the mortars produced with different percentages of AA of NSi, NS, CaCl and NC. Figure 3 shows the ratio of the f_cm_ strength of each mortar to that of the VA35 mortar at 7 d and 28 d. In addition, the strength increase (%) from 7 d to 28 d for each mixture produced with AA is shown.

The VA35-NSi1 (1 wt% NSi) mortars and VA35-CaCl2 (2 wt% CaCl) achieved the highest f_cm_ of 45 MPa and 41.3 MPa at 28 d, respectively. They reached a higher strength than VA35, at 37.6 MPa. In addition, from 7 to 28 d, the VA35-NSi1 and VA35-CaCl2 mortars increased by 28% and 20%, respectively (Figure 3). Moreover, these mortars also exhibited the highest early-age strength values, indicating that these alkaline activators accelerated the pozzolanic reactions. The presence of Cl^−^ in the activators may further accelerate PC. In contrast, the f_cm_ strengths of the mortars produced with 2% NSi and 4% CaCl activators reduced the f_cm_ strength values obtained with 1% NSi and 2% CaCl, respectively. This behaviour could be explained by an excess of Na^+^ due to the high alkali concentration in the pore solution and also partly due to the relatively weak binding (and exchangeability) of Na^+^ in the matrix, which remained unreacted in the mortar matrix and might have leached out [17].

Additionally, excess OH^−^ may prevent the dissolution of aluminosilicates and result in the formation of a material with lower strength [70]. For the CaCl activator, calcium ions (Ca^2+^) can provide a more alkaline environment for VA hydration, and chloride ions (Cl^−^) can participate in the reaction to generate crystals [27]. However, an excess activator concentration beyond the optimum value can decrease the strength because no more aluminium oxide is available for the reaction [33,71]. Farnam et al. (2015) [72] found that exposure of mortar samples to high-concentration CaCl_2_ solutions greater than approximately 11.3% (by mass) at room temperature of 23 °C resulted in oxychloride formation, leading to mortar degradation in less than 15 min. However, when corrosion is not considered an overwhelming concern, CaCl_2_ at low concentrations (1–2%) is still the chemical accelerator of choice due to its efficiency and the least expensive accelerator used in cementitious materials [73].

Regarding the mortars activated with NS, the VA35-NS2 mortar achieved a slightly higher compressive strength than the VA35 mortar after 7 days. Nevertheless, any concentration of the NS activator improved the f_cm_ strength of VA35 at 28 days. In addition, VA35-NS4 achieved a lower strength than VA35 mortar.

Similar to the NS activator mortar, the f_cm_ strength values of the VA35-NC mortars decreased when a higher percentage of the NC activator was employed. The VA35-NC2 mortar reached a 5% higher strength than the VA35 mortar at 28 d (Figure 3). The low mechanical performance of this set of mortars could be explained by the fact that NC is considered a weak activator because the reaction of the Ca^2+^ ions present in the precursor with the CO_3_^2−^ ions of the AA. This forming calcite, which occurs before the pH reaches the value necessary for the dissolution of silica and the formation of C-A-S-H gel, as observed in sodium carbonate-activated slag binders [74]. However, although the compressive strength of the control mortar was not achieved, the mortars activated with NC exhibited the most significant increase in strength (percentage) between days 7 and 28. This suggests that the VA35-NC binders exhibited slow but steady strength development over time.

During this phase, the activators NSi (1 wt%) and CaCl (2 wt%) potentially enhanced early-age strength development in VA mortars and achieved better mechanical performance at 28 days of all the activators evaluated. The subsequent experimental analysis was extended to a high replacement level of VA with the two selected activators.

- Phase 1.3: Mortar produced by adding lime as a corrector

**Figure 4:**
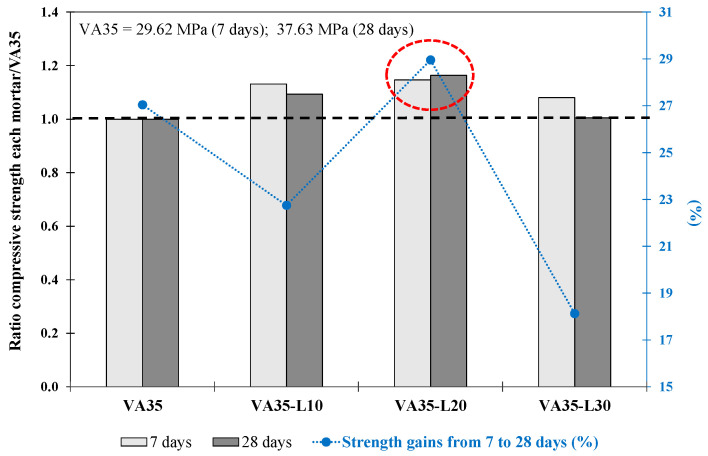
The ratio between compressive strength at 7 and 28 days of each mortar with respect to its corresponding VA35 mortar. The increase in strength from 7 d to 28 d (%) for the VA-based mortars produced with lime. The red circle describes the optimum mixture.

The mortars produced with up to 30% lime in replacement of VA (VA35-L10-30) achieved adequate and higher strength than the VA35 (untreated VA) and CVA35 (calcined VA) mortars at 7 days (see Table 5). In addition, at 28 days, the VA35-L10 and VA35-L20 mortars reached 41.1 MPa and 43.78 MPa, respectively, with a higher compressive strength than that of VA35 (37.63 MPa). Figure 4 shows the ratio of the f_cm_ strength of VA35-L with respect to its corresponding VA35 mortar strength at 7 and 28 days. At 28 days, VA35-L20 achieved a 16% higher strength value than VA35. Adding lime to silicon and aluminium oxides (within VA) with water causes pozzolanic reactions, forming C-S-H and C-A-H gels with a relatively strong structure [6]. In addition, Figure 4 shows the increased strength (%) from 7 to 28 days for each VA35-Ly mortar. At 7 d, the VA35-L10, VA35-L20, and VA35-L30 mortars achieved 81%, 78%, and 85%, respectively, of their f_cm_ strength at 28 d. As defined by Wang et al. [31], a lime-VA matrix improves the hydration rate and pozzolanic reactions of blended cement, consequently increasing its short-term strength. Among all the specimens, the VA35-L20 mortar exhibited the highest strength gain from days 7 to 28.

- Phase 1.4 and 1.5: Mortar produced using CVA, lime as corrector and alkali activator

**Figure 5:**
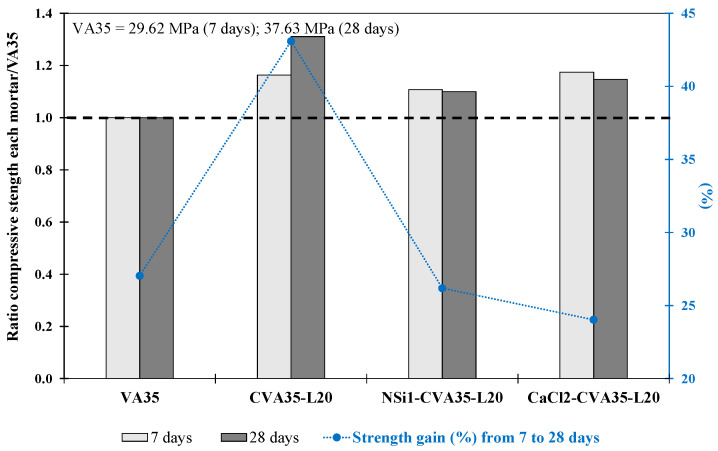
Compressive strength ratio at 7 and 28 days for each mortar with respect to its corresponding VA35 mortar. The increase in strength from 7 d to 28 d (%) for the VA-based mortars produced using the best activation methods.

The three mortars (CVA35-L20, CaCl2-CVA35-L20 and NSi1-CVA35-L20) produced in these phases were stronger than the VA35 mortar at 7 and 28 days (see Table 5 and Figure 5). The f_cm_ strength of the CVA35-L20 mortar was 31% higher, whereas the CaCl2-CVA35-L20 and NSi1-CVA35-L20 mortars achieved 15% and 10%, respectively, higher strength than that of the VA35 mortar at 28 days.

The CVA35-L20 mortar achieved the most significant strength gain (43%) from 7 to 28 d (see Figure 5), whereas the CaCl2-CVA35-L20 and NSi1-CVA35-L20 mortars achieved a strength gain of 23–27%. The addition of CVA and lime during the pozzolanic reaction was particularly beneficial for the strength gain of the mortars between days 7 and 28. According to the results obtained in this stage, the mortars produced with calcined VA at 700 °C for 1 h (phase 1.1), the use of 20 wt% lime (phase 1.3), and the activators CaCl (2 wt%) and NSi (1 wt%), in Phase 1.2, achieved the optimal strengths. The mortar produced by combining CVA and 20% lime achieved the highest mechanical performance (49.3 MPa). Furthermore, the mortars produced using 2 wt% CaCl2 with the CVA35-lime combination resulted in better performance than the VA35 mortar at 28 days. These findings were used for subsequent experiments on mortars containing 50% and 75% VA in Stage 2.

#### 3.2.2. Physical Properties

**Figure 6:**
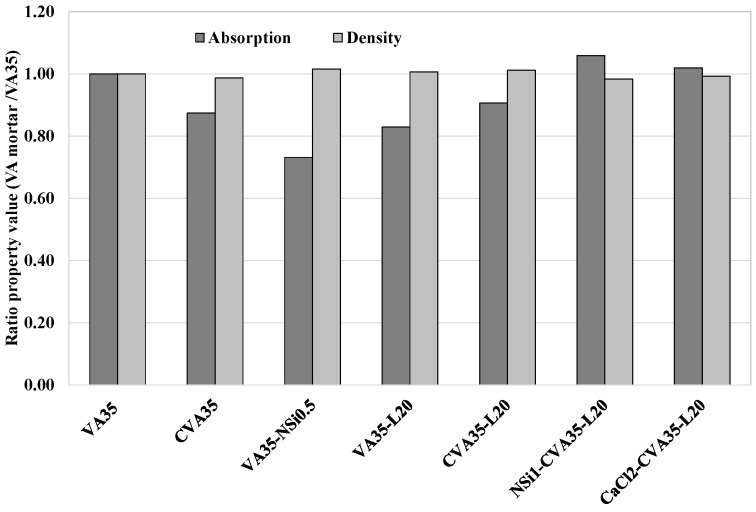
Ratio of Absorption capacity and density of VA35-based mortars with the best treatment methods with respect to that of control VA at 28 days.

Table 5 shows the dry bulk density, porosity, and water absorption results for all mortars produced with 35% VA and 65% PC after 7 and 28 days. The type of binder used under the applied pre-treatments significantly influenced the physical properties. However, all the optimal mixtures, defined according to their compressive strength values, achieved a similar density of 2.1 g/cc and 7–8% absorption. In addition, Figure 6 shows that the CVA35-700 mortar (Phase 1.1), which is mortar produced with calcined-treated VA, achieved lower water absorption and porosity values than the VA35 mortar. These findings suggest that calcined-treated VA contributed to a more advanced pozzolanic reaction and hydrated product formation resulting from the increased amorphous phase in the calcined VA in the cementitious composite (Figure 1).

According to the mortars produced with added lime as a corrector (Phase 1.3), the VA35-L20 and VA35-L30 mortars slightly increased the dry density and decreased the porosity compared to VA35 (see Table 5). The VA35-L20 mortar had 17% lower water absorption than the VA35 control mortar (see Figure 6).

The CVA35-L20-based mortars exhibited lower total porosity and water absorption as well as a higher density than the VA35 control mortar (see Table 5). A pozzolan-lime reaction happened, which formed more hydration products that filled the available pores, leading to a decrease in the porosity of the mortars [75].

Among the VA-based mortars produced using AAs (Phase 1.2), the VA35-CaCl2 mortar produced using 2 wt% of CaCl achieved an 8% lower porosity than VA35. Similarly, the VA35-NSi0.5 mortar, which incorporated 0.5 wt% of NSi, had the lowest water absorption and porosity values, resulting in up to a 27% reduction compared to the VA35 mortar (see Figure 6). Moreover, as shown in Table 5, VA35-NSi0.5 achieved a similar dry bulk density and higher f_cm_ strength (2.13 g/cc and 44 MPa) than VA35 mortar (2.10 g/cc and 37.6 MPa) at 28 d. These findings indicated that NSi formed a more compact and cohesive gel, which improved its physical properties and strength when used at low concentrations.

According to mortars produced using the three activation methods (Phase 1.5), the CaCl2-CVA35-L20 mortar achieved similar physical properties to VA35 with 15% higher compressive strength (see Table 5).

## 4. Stage 2 Mix Proportions: Results and Discussion

### 4.1. Mix Proportion of Mortars with 50% and 75% VA

During Stage 2 (see Table 6), mortars using 50% and 75% VA were produced, applying the most effective activation methods found in Stage 1. In Stage 2.1, mortars were produced with 50% VA, and four phases were carried out. In Stage 2.2, mortars were produced with 75% VA, and three phases were performed. The obtained results were compared with those of mortars made with 50% and 75% VA (without activation), named VA50 and VA75, respectively.

#### 4.1.1. Stage 2.1: Mix Proportion of Mortars with 50% VA

- Phase 2.1.1: Mortar produced using lime as a corrector

The exact three percentages (10%, 20% and 30%) of L used in Phase 1.3 were employed to replace VA in mortars produced with 50% VA. A 0.2 wt% SP (concerning binder weight) was used in all mortar mixtures. SP was added once the water was incorporated into the mixer. The mixtures were identified as *VA50-Ly*, where *y* denotes the %L.

- Phase 2.1.2: Mortar produced using CVA

Mortars were produced using 50% CVA (calcined at 700 °C for 1 h, defined in Phase 1.1). To achieve the desired workability, 0.4 wt% SP (concerning binder weight) was added to the mortar mixture. These mixtures were identified as *CVA50* based on the percentage of VA substitutions.

- Phase 2.1.3: Mortar produced using CVA and lime as a corrector

The mortar was produced using 50% CVA, and the optimal % of L was determined in Phase 2.1.1. To achieve adequate workability, 0.4 wt% SP (concerning binder weight) was added to the mortar mixture. These mortars were identified as *CVA50-Ly*, where *y* represents the %L used.

- Phase 2.1.4: Mortar produced using CVA, lime as corrector and AA

These mortars were prepared by combining CVA, the optimal lime % determined in Phase 2.1.1, and the most effective alkali activator tested in Phase 1.3. The mixture produced using NSi-activated required 0.2 wt% SP (concerning binder weight) to achieve adequate workability. The mixtures were identified as *AAz-CVAx-Ly*, where AA represents the alkaline activator type, *z* indicates the %AA concerning the binder weight, *x* denotes the %CVA used, and *y* denotes the %L replacement.

#### 4.1.2. Stage 2.2: Mix Proportion of Mortars with 75% VA

- Phase 2.2.1: Mortar produced using CVA

Mortars were produced using 75% CVA. Next, 0.4 wt% SP was added to the mixture. These mixtures were identified as *CVA75* based on the percentage of VA substitutions.

- Phase 2.2.2: Mortar produced using CVA and lime as a corrector

Mortars were produced using 75% CVA with 20% L; 0.4 wt% SP was added to the mixture. These mixtures were identified as *VA75-Ly*, where *y* denotes the %L (Table 6).

- Phase 2.2.3: Mortar produced using CVA, lime as corrector and AA

These mortars were tested by combining the 75% CVA, the optimal %L determined in Phase 2.1.1 (20% L), and the most effective AA tested in Phase 1.3. All mixtures required 0.6 wt% SP. The mixtures were identified as *AAz-CVAx-Ly*, where AA represents the alkaline activator type, *z* indicates the %AA concerning the binder weight, *x* indicates the %CVA used, and *y* denotes the %L replacement used.

### 4.2. Results and Discussion

#### 4.2.1. Compressive Strength (f_cm_)

The mortar produced with a higher amount of VA resulted in lower compressive strength. As shown in Table 7, the VA75 and VA50 mortars reached 24 MPa and 33 MPa, respectively, after 28 days.

##### Stage 2.1 Mix Proportion of Mortars with 50% VA

The results obtained for the mortars produced with lime as the corrector (Phase 2.1.1) are shown in Table 7. The VA50-L20 mortar reached 40 MPa at 28 days, which was significantly greater than that of the VA50 mortar. Conversely, the VA50-L30 mortar achieved a lower f_cm_ strength than the mortar made with 20% lime. Those results suggested that limited lime replacement exists to achieve the benefits of improved mortar performance. These findings were consistent with Donatello et al. (2010) [76], who recommended a 20% hydrated lime content for lime–pozzolan–cement mixtures. This result indicated that using 20 wt% L as a replacement was the best proportion when 50% replacement VA was used.

**Figure 7:**
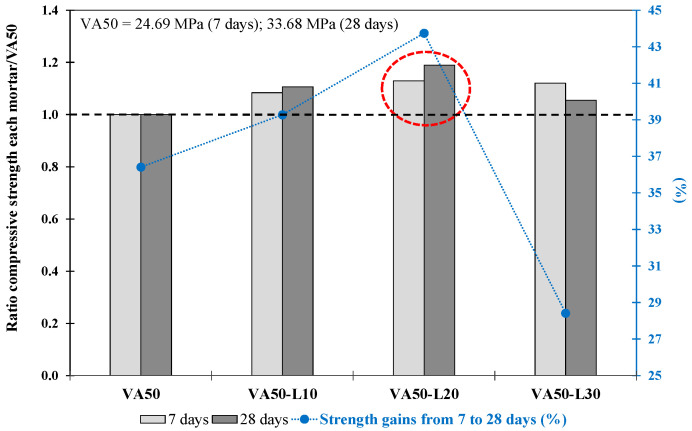
Compressive strength ratio at 7 and 28 days for each mortar with respect to its corresponding VA50 mortar. The increase in strength from 7 d to 28 d (%) of VA-based mortars produced with lime. The red circle describes the optimum mixture.

As shown in Figure 7, the VA50-L20 mortar exhibited the highest f_cm_ ratio values with respect to VA50 determined at 7 and 28 days. Furthermore, the VA50-L20 mortar exhibited up to 44% strength gain from 7 to 28 days, greater than the gains obtained by VA50, VA50-L10, and VA50-L30 mortars of 36%, 39%, and 28%, respectively. It is also worth noting that the strength gain of the 50% VA-lime-based mortar was higher than that of the 35% VA-lime-based mortars.

**Figure 8:**
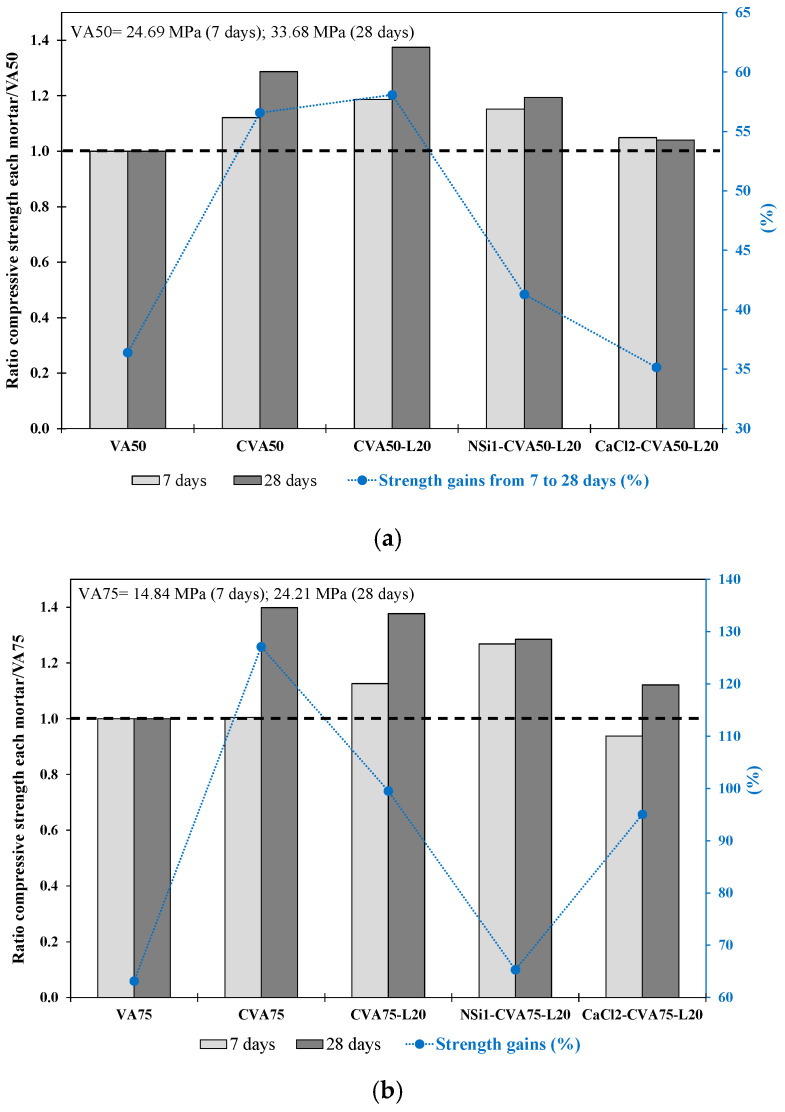
Ratio of compressive strength at 7 and 28 days for each mortar with respect to the (**a**) VA50 control mortar. (**b**) VA75 control mortar. The increase in strength from 7 d to 28 d (%) of the VA-based mortars produced using the best activation methods.

Table 7 shows (phases 2.1.2–2.1.4) that the mortar produced using calcined CVA or activators achieved higher f_cm_ strengths than those of the VA50 control mortar at 7 and 28 days. At 7 days, the CVA50-L20 mortar achieved the highest strength (29.2 MPa), followed by NSi1-CVA50-L20 (28.45 MPa). At 28 days, the CVA50-L20 achieved the highest strength, followed by the CVA50, NSi1-CVA50-L20 and CaCl2-CVA50-L20 mortars, which reached the f_cm_ strengths of 46.3 MPa, 43.3 MPa, 40.2 MPa and 35.02 MPa, respectively. The obtained values were 38%, 29%, 19% and 4% higher than those of VA50 mortar. In addition, CVA50 and CVA50-L20 achieved the highest strength increase, up to 58%, from 7 to 28 days (see Figure 8a). This increase in strength over time confirmed the positive impact of using CVA and the addition of lime to the binder. Furthermore, it is important to mention that the long-term strength increase of the 50% CVA-lime-based mortar was similar to that of the 35% VA replacement, confirming the positive effects of these treatments when the level of VA replacement increased.

Among the alkali-activated CVA50 mortars, while the CCl2-CVA50-L20 mortar reached similar f_cm_ strengths to those of the VA50 mortar at 7 and 28 days, the NSi1-CVA50-L20 mortar achieved better compressive strength values than those of the VA50. The NSi1-CVA50-L20 mortar increased its strength by 41% from 7 d to 28 d (see Figure 8a). In contrast, Erdoğan and Sağlık [77] concluded that the use of 2 wt% CaCl or NSi did not improve the strength of mortar produced with non-activated 50% perlite VA.

Based on f_cm_ results, among the mortars produced using 50% VA, the CVA50-L20 mortar achieved the highest strength, followed by the CVA50 and VA50-L20 mortars, which showed similar strength values to VA35 mortars. In addition, the mortar produced with 1 wt% NSi achieved the highest initial strength. At 28 days, 50% CVA-lime-based mortars activated with NSi achieved 40.2 MPa, 97% of the strength value obtained by 35% CVA-lime-based mortars activated with NSi mortars (41.4 MPa). These results also confirmed that, for a 50% VA replacement level, using NSi as an alkali activator in the binder improved its mechanical strength.

##### Stage 2.2 Mix Proportion of Mortars with 75% VA

Table 7 lists the properties of the mortars produced with 75% VA and 25% PC using the most effective activation methods defined in Stages 1 and 2.1. At 28 days, the CVA75 achieved the highest strength with 33.9 MPa, followed by CVA75-L20 with 33.3 MPa. The NSi1-CVA75-L20 and CaCl2-CVA75-L20 mortars also showed notable f_cm_ strengths, achieving 31.1 MPa and 27.2 MPa, respectively. These values were 40%, 38%, 29%, and 12% higher than those of the VA75 mortar.

Mortar made with 75% CVA (CVA75) and a 20 wt% lime addition (CVA75-L20) achieved the highest strength, 40% higher than that of the VA75 mortar, at 28 days. Furthermore, the CVA75 mortar achieved the highest strength gain of 127% between 7 and 28 days (Figure 8b). These results confirmed that calcination improved the properties of VA in terms of its amorphous composition. According to lime use, while the mortar produced with CVA 50 achieved the highest strength using 20 wt% lime, the CVA75 mortars required a higher percentage of lime. Other researchers have reported similar findings using natural pozzolans with high-volume replacements and lime percentages in the 20–30% range [15,64,78].

Similar to the VA50 mixtures, the alkali-activated CVA75-lime-based mortars achieved higher strengths than the VA75 mortar. However, the strength values obtained were lower than those of the non-alkali-activated mixtures. Nevertheless, the NSi1-CVA75-L20 mortar (using 1 wt% NSi) reached its highest strength at 7 days. Although the CaCl2-CVA75-lime-based mortars exhibited slightly lower strength in the earlier stages than the VA75 control mortars did, they exhibited a higher strength gain than the VA75- and NSi-CVA75-lime-based mortars at later stages (Figure 8b). Shi and Day [63] confirmed that adding 4% of CaCl_2_.2H_2_O reduced the early strength but improved the later strength of pastes containing 80% VA and 20% L cured at 23 °C. However, the measurement of free Ca(OH)_2_ in hardened pastes indicated that CaCl accelerated the consumption of Ca(OH)_2_ as the curing temperature increased above 35 °C, suggesting that the total pozzolanic reaction in CaCl-activated pastes was controlled by the dissolution of natural pozzolan at high temperatures. Other research works [38,79,80] also observed that mortars containing precursors with low calcium content, such as VA, were improved under moderate and high curing temperatures (40–90 °C).

Based on these findings, using calcined VA and lime, either separately or in combination, improved the mechanical performance of mortars with 75% VA replacement. However, the strength of the NSi-activated CVA75-lime-based mortars was weaker than that of the NSi-activated CVA50-lime-based mortars. Furthermore, according to previous research, heat curing can overcome the problems caused by slow strength gain owing to the high-VA content [77,81].

#### 4.2.2. Physical Properties

Generally, the VA-based mortars with 50% and 75% VA (Table 7) had higher porosities and water absorptions than those with 35% VA (Table 5). This can be attributed to the slower hydration rate with increasing VA content. The amorphous part of the aluminosilicate source may have participated in the reaction because of the substantial weight percentage of VA (50–75%), whereas the crystalline part remained unreacted. Additionally, the higher porosity could be related to the high concentrations of MgO and Fe_2_O_3_ present in the VA, which may not have participated in the hydration process in the cement. Studies have shown that the crystalline portion of Fe_2_O_3_ is not fully involved in the hydration process and remains unreacted [7]. Similarly, unreacted Mg from the VA content caused precipitation and decalcification of the C-S-H gels. It also interacted with the weaker magnesium-silicate-hydrate (MSH) phase, which showed increased porosity in the MSH phases [82].

**Figure 9:**
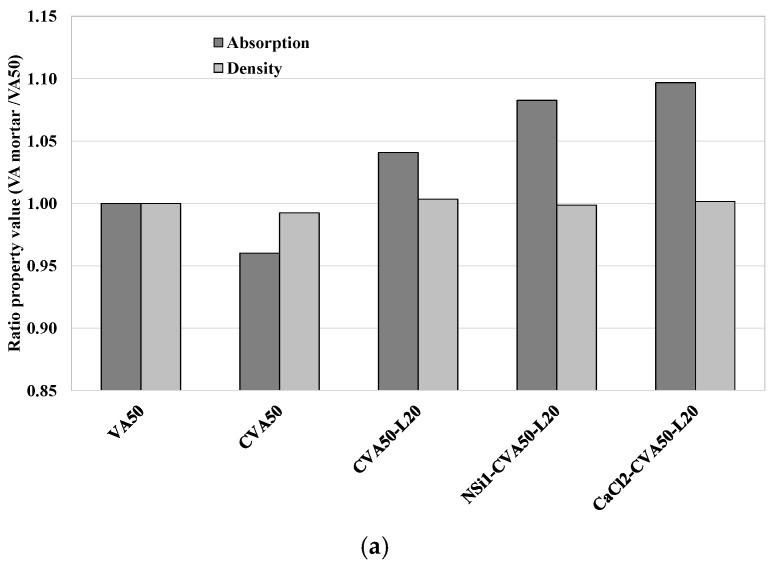

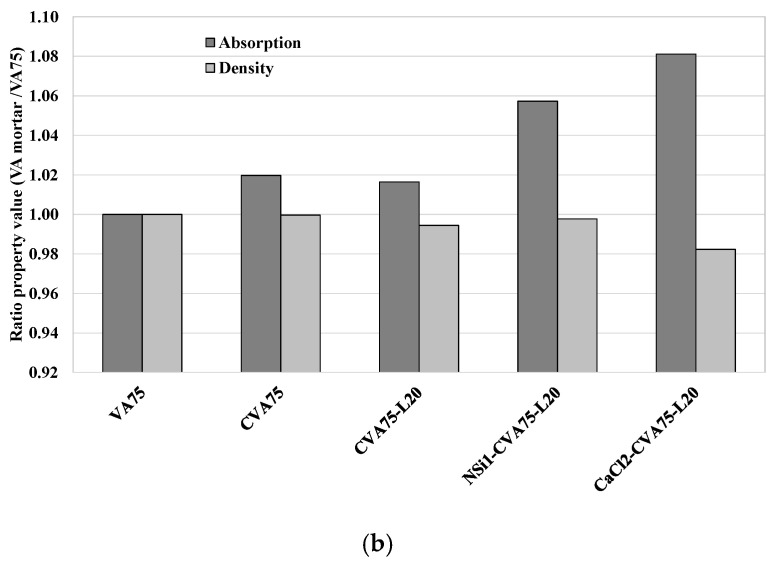
(**a**) Ratio of Absorption capacity and density of VA’-based mortars with the best treatment methods with respect to those of control VA at 28 days (**a**) V50 mortars. (**b**) V75 mortars.

Figure 9a shows the ratio of the physical properties (density and absorption) of VA mortar with respect to those of VA50 (control mixture) at 28 days. It shows that while the CVA50 achieved a lower absorption capacity, the AA mixtures achieved a higher absorption capacity that that of the VA50 mortar. Table 7 indicates that the porosity values ranged from 17% to 19.3%, and the densities of all samples were similar, from 2.06 to 2.08 g/cc. While the CVA50 achieved the lowest porosity, the AA mixtures achieved the highest. In addition, Table 7 shows that the porosity and water absorption of the control mortar VA50 decreased between 7 d and 28 d. In contrast, the opposite was observed in the other tested mortars but with slight variation. However, at this VA replacement level, additional curing days may be required to complete all pozzolanic reactions and lead to the action of unreacted VA, which also seals the space and reduces its porosity.

Figure 9b shows the ratio of the physical properties (density and absorption) of VA mortar with respect to those of VA75 (control mixture) at 28 days. It shows that all the activated samples achieved a higher absorption capacity than that of the VA75 mortar. 

Table 7 shows that the porosity values ranged from 18.6% to 19.4%. The density values of all samples were also similar, from 2.05 to 2.04 g/cc. The 75% VA-based mortars generally exhibited higher porosity, water absorption, and lower bulk density values than those of the 50% and 35% VA-based mortars. The fineness of VA plays a vital role in high-volume VA-based mortars, particularly due to its reduced fineness compared to PC. Since finer PC can fill the spaces between aggregates more effectively, replacing it with less fine VA or CVA could lead to poorer particle distribution, a more porous matrix and greater water absorption capacity.

Additionally, in mortar produced with high volume of VA, due to a lower density of VA than that of PC, a greater volume of VA was used as the PC substitution was done in weight. This fact was significant in 75% VA-based mortars, as the mortar specimens were composed of less sand and more paste, causing a reduction in density and an increase in absorption and porosity. In addition, Table 7 shows that using alkali activators in the CVA75-lime-based matrix did not contribute to better physical properties.

## 5. Conclusions

This study was based on a comprehensive experimental analysis of the compressive strength (f_cm_) and physical properties of VA-based mortars. The results led to several noteworthy conclusions.

- Calcined VA (CVA) at 700 °C for 1 h was the optimal thermal process, obtaining up to 59% of the amorphous component. In addition, the 20% lime in replacement of VA was optimal when the mortars were produced with 35–50% PC replacement.
- The CVA35-L20 and CVA50-L20 mortars achieved the highest compressive strength, with 49.3 MPa and 46.3 MPa, respectively. An increase of up to 40% compared to VA mortars. The absorption capacity was also reduced for 35–50% of untreated VA mortars.
- The effectiveness of calcined VA also showed that the CVA50 mortar achieved 43.5 MPa compressive strength, which was 29% higher than that of VA50. It also achieved lower absorption than that of VA50.
- Although the mortars made with 75% VA showed lower mechanical and physical properties compared to those with 35–50% VA. The mortars produced with 75% CVA and 20% lime achieved 33.9 MPa and 33.3 MPa, respectively, 40% higher than the mortars with 75% untreated VA.
- Although the mortars using alkali activators (AA) achieved a lower compressive strength at 28 days than those produced with CVA and lime addition, the mortars using 2% CaCl or 1% NSi activator and 35% of untreated VA achieved the highest strength at 7 days (34.4 MPa and 35.6 MPa, respectively). Moreover, at 28 days, the mortars produced with 2% CaCl or 1% NSi activators achieved a strength of 41 MPa and 45 MPa, respectively, avoiding using the calcination process in the VA activation.
- The use of AA in mortars with 50–75% CVA and lime did not improve the properties of mortars CVA-lime at 28 days. However, the NSi-activated CVA-lime-based mortar with 75% CVA achieved 13% higher compressive strength at 7 days compared to the CVA-lime mortars.

This study highlighted the applicability of up to 50% VA in PC replacement. However, the study also highlighted the need for more extended curing periods under standard conditions (25 °C, 95% RH) to ensure complete pozzolanic reactions and optimal mechanical properties; in addition, further research is required to determine the exact curing requirements for these high-VA mortars.

## Figures and Tables

**Table 1:** Chemical composition of volcanic ash (VA), calcined VA (CVA), portland cement (PC) and lime (L) by weight of oxides (%).

(%)	SiO_2_	Al_2_O_3_	CaO	Fe_2_O_3_	MgO	SO_3_	Na_2_O	K_2_O	Others	LOI
VA	60.15	16.51	6.30	6.22	3.26	0.03	3.62	1.17	0.94	1.81
PC	19.4	4.2	63.5	3.4	1.4	3.0	0.12	0.53	-	3.7
Lime	1.32	0.66	88.8	0.26	2.2	-	-	-	1.0	-

**Table 2:** Chemical properties requirements of VA according to ASTM C618 [50].

Requirements	Class N, ASTM C618	VA
SiO_2_ + Al_2_O_3_ + Fe_2_O_3_, %	Min, 70.0	82.88
SO_3_, %	Max, 4.0	0.0312
Moisture content, %	Max, 3.0	6.5
Loss of ignition, %	Max, 10.0	1.81

**Table 3:** Released metal concentration after EN 12457-2 leaching test at L/S = 10 L/kg.

Metals	VA (mg/kg)	EN 12457-2		
		Inert (mg/kg)	Stable Non-Reactive (mg/kg)	Hazardous (mg/kg)
Na	613.04	-		
Si	9.52	-		
Cr	6.51	0.5	10	70
Ni	0.02	0.4	10	40
Mo	0.28	0.5	10	30
Cu	0.02	2	50	100
Cd	ND	0.04	1.0	5.0
Sb	ND	0.06	0.7	5.0
As	ND	0.5	2.0	25
Zn	ND	4.0	50	200
Se	ND	0.1	0.5	7
Pb	ND	0.5	10	50
Ba	10.54	20	100	300
Hg	ND	0.01	0.2	2

**Table 4:** Description of mortar mixes related to Stage 1—Validation of activation method. Mixtures that require superplasticiser (SP) are denoted by (*).

Mix	Description
Phase 1.1: Calcined VA (CVA)	
CVA35-500	35%CVA + 65%PC
CVA35-700	35%CVA + 65%PC
CVA35-800	35%CVA + 65%PC
CVA35-900	35%CVA + 65%PC
Phase 1.2: Alkali-activators	
VA35-NSi2	(35%VA + 65%PC) + 2%Na_2_SiO_3_
VA35-NSi1	(35%VA + 65%PC) + 1% Na_2_SiO_3_
VA35-NSi0.5	(35%VA + 65%PC) + 0.5% Na_2_SiO_3_
VA35-CaCl4 *	(35%VA + 65%PC) + 4%CaCl_2_ (0.2%SP)
VA35-CaCl2	(35%VA + 65%PC) + 2%CaCl_2_
VA35-CaCl1	(35%VA + 65%PC) + 1%CaCl_2_
VA35-NS4 *	(35%VA + 65%PC) + 4%Na_2_SO_4_ (0.4%SP)
VA35-NS2	(35%VA + 65%PC) + 2% Na_2_SO_4_
VA35-NC4 *	(35%VA + 65%PC) + 4%Na_2_CO_3_ (0.2%SP)
VA35-NC2	(35%VA + 65%PC) + 2% Na_2_CO_3_
Phase 1.3: Lime as corrector	
VA35-L10	35% (90%VA + 10%L) + 65%PC
VA35-L20	35% (80%VA + 20%L) + 65%PC
VA35-L30	35% (70%VA + 30%L) + 65%PC
Phase 1.4: CVA and lime as corrector	
CVA35-L20	35% (80%CVA + 20%L) + 65%PC
Phase 1.5: CVA, lime as corrector and alkali-activator	
NS1-CVA35-L20	35% (80%CVA + 20%L) + 65%PC + 1%Na_2_SiO_3_
CaCl2-CVA35-L20	35% (80%CVA + 20%L) + 65%PC + 2%CaCl_2_
Control mixture	
VA35	35%VA + 65%PC

**Table 5:** Compressive strength values with the standard deviation in brackets and the physical properties of all mixes related to Stage 1. Mixtures that require superplasticiser (SP) are denoted by (*).

Mix	Compressive Strength (MPa)		Porosity (%)		Water Absorption (%)		Dry Bulk Density (g/cc)	
	7 d	28 d	7 d	28 d	7 d	28 d	7 d	28 d
CVA35-500	28.64 (1.1)	42.76 (1.2)						
CVA35-700	30.58 (0.56)	44.50 (0.3)	17.31	14.59	8.43	7.06	2.052	2.068
CVA35-800	29.84 (0.80)	43.60 (1.2)						
CVA35-900	27.41 (1.40)	41.38 (1.0)						
VA35-NSi2	32.17 (0.11)	41.36 (0.67)	13.78	13.90	6.58	6.58	2.094	2.112
VA35-NSi1	35.64 (1.16)	44.99 (0.99)	14.58	14.26	6.96	6.86	2.096	2.084
VA35-NSi0.5	33.84 (1.45)	44.17 (2.09)	15.99	12.57	7.69	5.91	2.081	2.129
VA35-CaCl4 *	27.91 (1.02)	40.57 (1.45)	16.93	16.29	8.24	7.93	2.057	2.054
VA35-CaCl2	34.27 (1.18)	41.29 (0.89)	14.38	15.56	6.88	7.46	2.090	2.087
VA35-CaCl1	33.82 (1.06)	40.40 (0.41)	17.09	17.14	8.36	8.22	2.044	2.085
VA35-NS4 *	27.93 (1.10)	36.93 (1.50)	16.06	13.17	7.75	6.30	2.078	2.128
VA35-NS2	30.79 (2.00)	37.50 (2.26)	13.79	12.89	6.51	6.04	2.119	2.133
VA35-NC4 *	18.63 (0.35)	28.59 (0.48)	16.69	13.70	8.05	6.53	2.076	2.100
VA35-NC2	25.69 (0.70)	39.66 (3.49)	14.33	14.88	6.82	7.09	2.101	2.100
VA35-L10	33.5 (0.17)	41.12 (1.93)	17.87	16.77	8.62	8.04	2.071	2.085
VA35-L20	33.95 (1.31)	43.78 (2.51)	14.06	14.11	6.70	6.69	2.100	2.109
VA35-L30	32.0 (2.03)	37.80 (2.10)	18.12	14.07	8.88	6.64	2.041	2.122
CVA35-L20	34.48 (0.77)	49.31 (0.96)	17.74	15.52	8.47	7.32	2.096	2.121
NS1-CVA35-L20	32.80 (1.34)	41.39 (1.82)	17.98	17.60	8.73	8.55	2.059	2.062
CaCl2-CVA35-L20	34.79 (3.04)	43.15 (2.32)	17.20	17.12	8.29	8.23	2.075	2.080
VA35	29.62 (2.43)	37.63 (2.46)	17.56	16.92	8.44	8.07	2.080	2.096

**Table 6:** Description of mortar mixes related to Stage 2—Validation of high-volume VA use in mortar. Mixtures that require superplasticiser (SP) are denoted by (*).

Mix	Description
Stage 2.1 mortar produced with 50% VA	
Phase 2.1.1: Lime as corrector	
VA50-L10 *	50% (90%VA + 10%L) + 50%PC (0.2%SP)
VA50-L20 *	50% (80%VA + 20%L) + 50%PC (0.2%SP)
VA50-L30 *	50% (70%VA + 30%L) + 50%PC (0.2%SP)
Phase 2.1.2: CVA	
CVA50 *	50% CVA + 50% PC (0.4% SP)
Phase 2.1.3: CVA and lime as corrector	
CVA50-L20 *	50% (80%CVA + 20%L) + 50%PC (0.4%SP)
Phase 2.1.4: CVA, lime corrector and alkali-activator	
NSi1-CVA50-L20 *	50% (80%CVA + 20%L) + 50%PC +1%Na_2_SiO_3_ (0.2%SP)
CaCl2-CVA50-L20	50% (80%CVA + 20%L) + 50%PC + 2%CaCl_2_
Control mixture	
VA50 *	50% VA + 50% PC (0.2%SP)
Phase 2.2: mortar produced with 75% VA	
Phase 2.2.1: CVA	
CVA75 *	75% CVA + 25% PC (0.4%SP)
Phase 2.2.2.: CVA and lime as corrector	
CVA75-L20 *	75% (80%CVA + 20%L) + 25%PC +1% Na_2_SiO_3_ (0.4%SP)
Phase 2.2.3.: CVA, lime corrector and alkali-activator	
NSi1-CVA75-L20 *	75% (80%CVA + 20%L) + 25%PC +1% Na_2_SiO_3_ (0.6%SP)
CaCl2-CVA75-L20 *	75% (80%CVA + 20%L) + 25%PC +2%CaCl_2_ (0.6%SP)
Control mixture	
VA75 *	70% VA + 25% PC (0.2%SP)

**Table 7:** Compressive strength values with the standard deviation in brackets and the physical properties of all mixes related to Stage 2. Mixtures that require superplasticiser (SP) are denoted by (*).

Mix	Compressive Strength (MPa)		Porosity (%)		Water Absorption (%)		Dry Bulk Density (g/cc)	
	7 d	28 d	7 d	28 d	7 d	28 d	7 d	28 d
Stage 2.1 mortar produced with 50% VA								
VA50-L10 *	26.76 (0.67)	37.27 (2.93)	18.33	17.74	9.01	8.67	2.034	2.047
VA50-L20 *	27.87 (1.80)	40.06 (1.72)	18.22	18.47	8.94	9.04	2.037	2.043
VA50-L30 *	27.66 (0.98)	35.52 (2.97)	17.91	15.51	8.74	7.52	2.050	2.067
CVA50 *	27.68 (0.34)	43.34 (2.55)	16.49	17.03	8.08	8.26	2.041	2.061
CVA50-L20 *	29.29 (1.70)	46.31 (3.76)	17.53	18.66	8.47	8.96	2.070	2.084
NSi1-CVA50-L20 *	28.45 (0.82)	40.20 (0.19)	18.07	19.32	8.78	9.32	2.059	2.074
CaCl2-CVA50-L20	25.91 (2.22)	35.02 (1.59)	16.93	19.63	8.15	9.44	2.077	2.080
Control mixture								
VA50 *	24.69 (0.95)	33.68 (2.45)	18.38	17.87	9.03	8.60	2.036	2.077
Stage 2.2 mortar produced with 75% VA								
CVA75 *	14.91 (0.93)	33.86 (1.57)	18.60	18.80	9.26	9.18	2.008	2.048
CVA75-L20 *	16.71 (0.90)	33.34 (1.72)	18.64	18.62	9.26	9.15	2.013	2.037
NSi1-CVA75-L20 *	18.82 (0.84)	31.11 (1.71)	18.83	19.46	9.33	9.52	2.018	2.044
CaCl2-CVA75-L20 *	13.92 (0.78)	27.15 (1.68)	18.67	19.59	9.27	9.77	2.014	2.013
VA75 *	14.84 (3.22)	24.21 (0.21)	18.40	18.45	9.07	9.00	2.028	2.049

## Data Availability

Data are contained within the article.
